# Comprehensive Proteomics Metadata and Integrative Web Portals Facilitate Sharing and Integration of LINCS Multiomics Data

**DOI:** 10.1016/j.mcpro.2025.100947

**Published:** 2025-03-13

**Authors:** Dušica Vidović, Behrouz Shamsaei, Stephan C. Schürer, Phillip Kogan, Szymon Chojnacki, Michal Kouril, Mario Medvedovic, Wen Niu, Evren U. Azeloglu, Marc R. Birtwistle, Yibang Chen, Tong Chen, Jens Hansen, Bin Hu, Ravi Iyengar, Gomathi Jayaraman, Hong Li, Tong Liu, Eric A. Sobie, Yuguang Xiong, Matthew J. Berberich, Gary Bradshaw, Mirra Chung, Robert A. Everley, Ben Gaudio, Marc Hafner, Marian Kalocsay, Caitlin E. Mills, Maulik K. Nariya, Peter K. Sorger, Kartik Subramanian, Chiara Victor, Maria Banuelos, Victoria Dardov, Ronald Holewinski, Danica-Mae Manalo, Berhan Mandefro, Andrea D. Matlock, Loren Ornelas, Dhruv Sareen, Clive N. Svendsen, Vineet Vaibhav, Jennifer E. Van Eyk, Vidya Venkatraman, Steve Finkbiener, Ernest Fraenkel, Jeffrey Rothstein, Leslie Thompson, Jacob Asiedu, Steven A. Carr, Karen E. Christianson, Desiree Davison, Deborah O. Dele-Oni, Katherine C. DeRuff, Shawn B. Egri, Alvaro Sebastian Vaca Jacome, Jacob D. Jaffe, Daniel Lam, Lev Litichevskiy, Xiaodong Lu, James Mullahoo, Adam Officer, Malvina Papanastasiou, Ryan Peckner, Caidin Toder, Joel Blanchard, Michael Bula, Tak Ko, Li-Huei Tsai, Jennie Z. Young, Vagisha Sharma, Ajay Pillai, Jarek Meller, Michael J. MacCoss

**Affiliations:** 1 BD2K-LINCS DCIC, Department of Molecular and Cellular Pharmacology, University of Miami, Miami, Florida, USA; 2 Sylvester Comprehensive Cancer Center, Miller School of Medicine, University of Miami, Miami, Florida, USA; 3 BD2K-LINCS DCIC, Department of Environmental and Public Health Sciences, University of Cincinnati, Cincinnati, Ohio, USA; 4 BD2K-LINCS DCIC, Frost Institute for Data Science & Computing, University of Miami, Miami, Florida, USA; 5 DToxS, Department of Pharmacological Sciences, Icahn School of Medicine at Mount Sinai, New York, New York, USA; 6 DToxS, Center for Advanced Proteomics Research, Rutgers University New Jersey Medical School, Newark, New Jersey, USA; 7 HMS LINCS Center, Harvard Medical School, Boston, Massachusetts, USA; 8 NeuroLINCS, Cedars-Sinai Medical Center, Los Angeles, California, USA; 9 NeuroLINCS, Gladstone Institute of Neurological Disease and the Departments of Neurology and Physiology, University of California San Francisco, San Francisco, California, USA; 10 NeuroLINCS, Department of Biological Engineering, MIT, Cambridge, Massachusetts, USA; 11 NeuroLINCS, Department of Neuroscience, Johns Hopkins University, Baltimore, Maryland, USA; 12 NeuroLINCS, Departments of Psychiatry and Human Behavior and Neurobiology and Behavior, University of California Irvine, Irvine, California, USA; 13 PCCSE, The Broad Institute of Harvard and MIT, Cambridge, Massachusetts, USA; 14 PCCSE, Picower Institute for Learning and Memory, Department of Brain and Cognitive Sciences, Massachusetts Institute of Technology, Cambridge, Massachusetts, USA; 15 PCCSE, Department of Genome Sciences, University of Washington, Seattle, Washington, USA; 16 NIH, Bethesda, Maryland, USA

**Keywords:** LINCS proteomics metadata, metadata harmonization, FAIRness, P100 data, LINCS data portal, piNET

## Abstract

The Library of Integrated Network-based Cellular Signatures (LINCS), an NIH Common Fund program, has cataloged and analyzed cellular function and molecular activity profiles in response to >80,000 perturbing agents that are potentially disruptive to cells. Because of the importance of proteins and their modifications to the response of specific cellular perturbations, four of the six LINCS centers have included significant proteomics efforts in the characterization of the resulting phenotype. This manuscript aims to describe this effort and the data harmonization and integration of the LINCS proteomics data discussed in recent LINCS papers.

## Highlights

• The Library of Integrated Network-Based Cellular Signatures (LINCS) has diverse data.
• Targeted proteomics, data-dependent/independent acquisition technologies utilized.
• Metadata standards harmonize data and ensure compatibility with community platforms.
• LINCS tools make proteomics signatures accessible for exploration and analysis.

## Graphical Abstract

**Figure:**
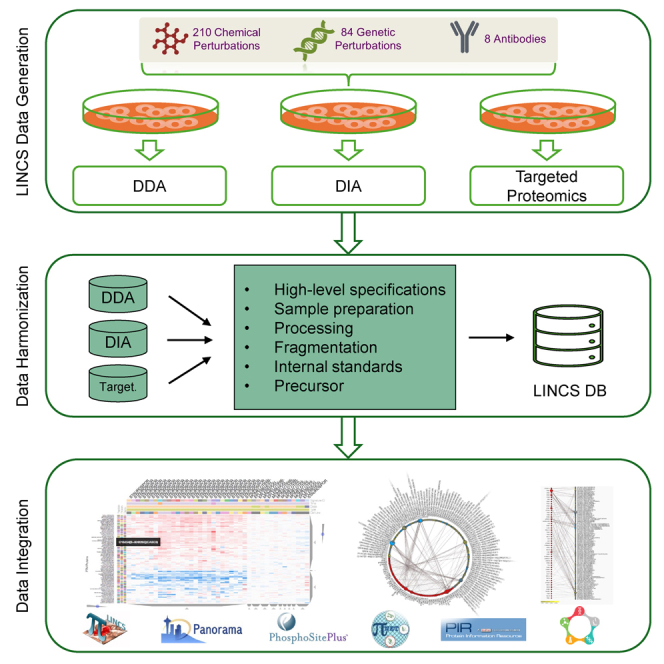

## In Brief

The Library of Integrated Network-Based Cellular Signatures (LINCS) built a FAIR-aligned library of cellular response signatures to support disease research and drug discovery. LINCS proteomics data were generated at four centers using diverse technologies like P100, tandem mass tags, and SWATH to study cancer, cardiotoxicity, and neurodegenerative diseases. Metadata standards were developed to harmonize data and ensure compatibility with community platforms. LINCS tools, such as the Data Portal and piNET, make these proteomics signatures easily accessible for exploration and analysis.

## Overview of the LINCS Program

The Library of Integrated Network-Based Cellular Signatures (LINCS) program has created an extensive reference library of cell-based perturbation response signatures incorporating a large number of perturbagens, model systems, and assays. The project is based on the premise that disrupting any one of the many steps of a given biological process will cause related changes in the molecular and cellular characteristics, behavior, and/or function of the cell—the observable composite of which is known as the cellular phenotype. Observing how and when a cell’s phenotype is altered by specific stressors can provide clues about the underlying mechanisms involved in perturbation and, ultimately, disease. To best characterize this cellular phenotype, it is important to include protein-based signatures - the primary functional macromolecules in the cell.

LINCS data have been made openly available as a community resource through a series of data releases to enable scientists to address a broad range of basic research questions. Results were obtained in cultured and primary cells whose state has been perturbed experimentally, with the term “perturbagen” used to refer to any condition that can alter the cellular state. LINCS datasets therefore consist of assay results from cells treated with bioactive small molecules, antibodies, and ligands, such as growth factors and cytokines, microenvironment proteins, genetic perturbations, and comparisons of disease versus normal primary cells from patients and healthy control subjects. The data production effort of the LINCS program began in 2014 and ended in 2020. The program involved the production of perturbation-induced molecular and cellular signatures from six Data and Signature Generation Centers (DSGCs). The data have been released as a community resource with metadata annotations that strictly follow the Findable, Accessible, Interoperable, and Reusable (FAIR) guidelines.

Because of the importance of proteins and their modifications to the response of specific cellular perturbations, four of the six LINCS centers have included significant proteomics efforts in the characterization of their samples. In addition to the four data production sites, there was also a significant effort by a Data Coordination and Integration Center (DCIC) to synergize the efforts between the LINCS sites and the NIH Big Data to Knowledge (BD2K) program. Additionally, the personnel performing proteomics data production formed an inter-center Proteomics Working Group (PWG) to discuss shared needs across the LINCS program and to promote outreach efforts to improve the visibility of the proteomics data production efforts within the program.

**Fig. 1:**
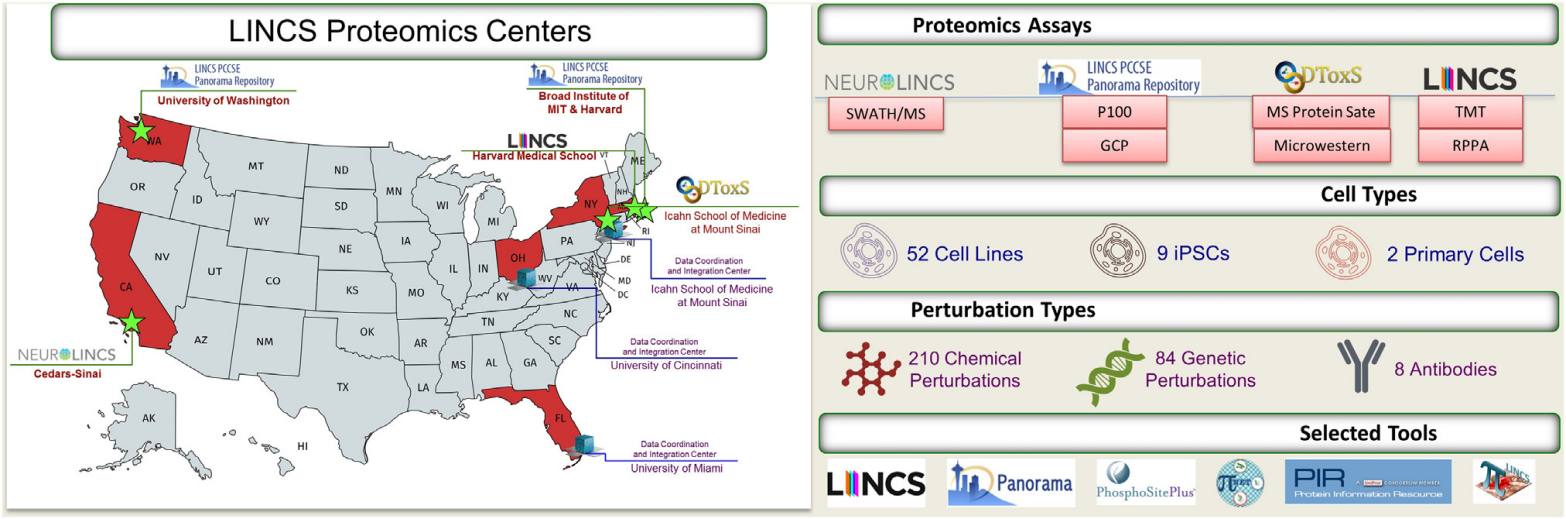
**Overview of multi-institutional LINCS proteomics data centers and assays**. On the *left*, the list of participating centers in LINCS proteomics working group: BD2K-LINCS DCIC: Department of Molecular and Cellular Pharmacology, University of Miami, Miami, FL 33146; Department of Environmental and Public Health Sciences, University of Cincinnati, Cincinnati, OH 45220; Department of Pharmacological Sciences, Icahn School of Medicine at Mount Sinai, New York, NY 10029, DToxS: Department of Pharmacological Sciences, Icahn School of Medicine at Mount Sinai, New York, NY 10029, HMS LINCS Center: Harvard Medical School, Boston, MA 02115, NeuroLINCS: Cedars-Sinai Medical Center, Los-Angeles, CA 90048; Gladstone Institute of Neurological Disease and the Departments of Neurology and Physiology, University of California San Francisco, San Francisco, CA 94158; Department of Biological Engineering, MIT, Cambridge, MA 02142; Department of Neuroscience, Johns Hopkins University, Baltimore, MD 21205; Departments of Psychiatry and Human Behavior and Neurobiology and Behavior, University of California Irvine, Irvine, CA 92697, PCCSE: The Broad Institute of Harvard and MIT, Cambridge, MA 02142; Department of Genome Sciences, University of Washington, Seattle, WA 98195; Picower Institute for Learning and Memory, Department of Brain and Cognitive Sciences, Massachusetts Institute of Technology, Cambridge, MA 02139. On the *right*, the graphical summary of LINCS proteomics assays, cell lines, and perturbation types profiled as part of LINCS and described in this paper, as well as LINCS-related tools and resources providing platforms for proteomics data annotation and sharing: piNET (1), Panorama (2, 3), phosphoSitePlus (4, 5), The Protein Information Resource (6), and HMS LINCS (7).

This manuscript aims to provide an overview of the LINCS proteomics data, data harmonization, and data integration for the data published by the four LINCS proteomics centers. Figure 1 shows the overview of the LINCS proteomics data centers, along with the utilized proteomics assays and integration tools developed by the centers, such as piNET (1), Panorama (2, 3), phosphoSitePlus (4, 5), The Protein Information Resource (6), and HMS LINCS (7).

## Challenges with Proteomics Data Integration

Proteomics data integration plays a critical role in advancing our understanding of complex biological systems. By synthesizing diverse proteomic datasets, integration enables the elucidation of protein functions, interactions, and regulatory mechanisms, offering comprehensive insights into cellular processes.

While the inclusion of proteomics is obvious, the diversity of proteomics technologies and assays made the proteomics data integration a particularly significant challenge for a large data production effort. Ultimately the four proteomics groups from four LINCS data production centers settled on five different assay types chosen based on the expertise and instrumentation available with the respective groups. These data included the use of targeted data analysis (GCP and P100 assays), data collected by data dependent acquisition using tandem mass tags and spectrum counting, and data independent acquisition (SWATH). While the datasets within assay types are very valuable, the harmonization of quantitative proteomics data between assay types has yet to be solved.

To facilitate proteomics data dissemination and integration, several resources have been developed and established such as ProteomicsDB (8), PRIDE (9), and DeepCoverMOA (10).

ProteomicsDB is a comprehensive platform that integrates large-scale proteomic datasets across multiple organisms. It harmonizes quantitative data on protein expression, post-translational modifications, and protein-protein interactions from various biological contexts. The integration tools within ProteomicsDB support hypothesis generation and validation, with powerful data visualization and querying features that enable researchers to explore and analyze proteomic data from multiple studies. However, its primary strength lies in the integration of data across various experimental setups rather than data across different omics layers.

PRIDE serves as a central repository for proteomics data, offering an open-access platform for sharing, integrating, and reanalyzing proteomics datasets. PRIDE's integration methods focus on facilitating cross-study data comparisons and enhancing data transparency and reproducibility. It allows researchers to share raw mass spectrometry data, protein identifications, and peptide sequences in standardized formats, making it a pivotal resource for the proteomics community (11). The reanalysis capability of PRIDE fosters integrative studies, enabling novel insights from previously published datasets.

DeepCoverMOA focuses on integrating proteomics data to elucidate mechanisms of action (MOA) in drug discovery. Combining proteomics data with other molecular and clinical data aids in identifying therapeutic targets and understanding drug effects at the molecular level. The integration methodologies employed by DeepCoverMOA are aimed at refining the drug development process, particularly through the identification of biomarkers and drug-protein interactions.

The LINCS proteomics data are integrable with these resources via a set of standardized data and reagent representations, such as proteins names and systematic accession IDs, Ensembl gene symbols, and tissue or drug names. In addition, as part of the LPLP, we have integrated the exploration of proteomic signatures associated with compounds analyzed in the DeepCoverMOA initiative. For example, when searching for a compound that exists in DeepCoverMOA, in addition to exploring LINCS signatures, the user will be guided to explore the DeepCoverMOA compound signature in Harmonizome (12, 13).

## Diverse Proteomics Assay Types and Applications

The Broad Institute's LINCS Proteomic Characterization Center for Signaling and Epigenetics (PCCSE) (14) aimed to understand how changes in cellular signaling, transcriptional, and epigenetic states influence one another via feedforward and feedback processes. Perturbations to these states were induced by drug treatments or genetic manipulations (via CRISPR) of cancer cell models, neuronal lineages, or primary vascular cells. The PCCSE used mass spectrometry-based targeted proteomics to assay cellular phosphosignaling (P100 assay) and histone modification (GCP) responses elicited by these perturbations (15). These experiments, coupled with matched L1000 data obtained via collaboration with the LINCS Center for Transcriptomics, were designed to test the hypothesis that early cell signaling responses to perturbation may establish new cellular states by altering epigenetic landscapes. Data from both the P100 and the GCP assays are compatible with the CMAP framework (16) developed by the LINCS Center for Transcriptomics to interrogate L1000 data.

The Harvard Medical School (HMS) LINCS Center quantitative proteomics (17) was based on tandem mass tags to collect data and compute signatures from cells exposed to small-molecule drugs and naturally occurring ligands. The Center emphasized the integration of their mass spectrometry data with their imaging and transcript profiling approaches. The Center also developed data analysis pipelines and informatics systems, which are particularly important in the case of microscopy data for which few such systems exist. The research products of the HMS Center comprise new measurement technologies, multi-dimensional datasets, and open-source software.

The Drug Toxicity Signature (DToxS) Generation LINCS Center at the Icahn School of Medicine at Mount Sinai in New York aimed to generate cellular signatures that predict adverse effects of Food and Drug Administration (FDA)-approved drugs, with a focus on cardiotoxicity (18). The efforts of DToxS have been focused on cardiotoxicity caused by cancer therapeutics, particularly heart failure and reduced ventricular function resulting from treatment with kinase inhibitors. The DToxS Center used proteomic measurements conducted at the Center for Advanced Proteomics at Rutgers New Jersey Medical School. Proteins were quantified by spectral counts, which is a widely accepted semi-quantitative, label-free estimate of protein levels. Different levels of data from raw spectral counts to ranked lists of drug-dependent differentially expressed proteins, and the subcellular processes the differentially expressed proteins participate in, are available for each drug in each of the tested cell lines.

The NeuroLINCS Center studied the motor neuron diseases amyotrophic lateral sclerosis (ALS) and spinal muscular atrophy (SMA) by characterizing the molecular networks within patient-derived induced pluripotent stem cells (iPSCs) and their differentiated motor neuron progeny (19). However, the NeuroLINCS Center was expanded to develop a global cortical neuron assay from iPSCs that will be of interest to groups working in other neurodegenerative diseases such as Alzheimer's disease. The proteomics assay from NeuroLINCS was based on SWATH mass spectrometry (20) to inspect differences in the proteomic landscapes of ALS and SMA patient neurons compared with unaffected controls. Perturbations included ALS- and SMA-relevant mutations in patient-derived iPSCs as well as additional chemical perturbations. NeuroLINCS has been a collaborative effort between multiple research groups at the University of California, Irvine, Cedars-Sinai Medical Center, the Gladstone Institute, MIT, and Johns Hopkins University.

## Development and Harmonization of the LINCS Proteomics Metadata towards FAIR Standards

The LINCS consortium strived to adhere to the key FAIR guiding principles - Findability, Accessibility, Interoperability, and Reusability (21). To facilitate the FAIRness of the LINCS generated data, we have: (i) established the data and reagent categories; (ii) developed the reporting standards of data and metadata, (iii) integrated community standardized formats, controlled vocabularies, ontologies, and external identifiers for the entities of interest, and (iv) developed a publicly accessible data portal.

As one of the primary goals of the LINCS project has been the data integration and analysis of diverse datasets, it is essential that the data and metadata reporting standards sufficiently describe the assays and screening results. The metadata standards development requires defining which biological entities and concepts, experimental parameters, and results must be reported. The LINCS metadata standards are available to access through the LINCS project website (https://lincsproject.org/LINCS/data/standards), in addition to being published in FAIRsharing (https://fairsharing.org/3532).

To develop the experimental metadata standards for the LINCS proteomics data, the assays were organized into three categories: (1) data-dependent acquisition (DDA) that co22rresponds to the discovery of proteomics data, (2) the data-independent acquisition (DIA) that corresponds to the LINCS P100 and SWATH assays, and (3) the targeted proteomics data that corresponds to the GCP assay. For each of the LINCS proteomics data types, the rich experimental metadata standards specifications have been developed by reviewing the community-established minimum information specifications related to reagents and assays (such as Minimum Information About a Bioactive Entity (MIABE) (22) and Minimum Information About a Cellular Assay (MIACA) (https://miaca.sourceforge.net/)) as well as proteomics data focused specifications (such as NCI Proteomics Data (https://proteomic.datacommons.cancer.gov/pdc/), CPTAC (23) and PRIDE (9) repositories). The input specific to the LINCS assays from the individual DSGCs (DToxS, HMS LINCS, NeuroLINCS, and PCCSE, described above) was also collected. This effort was led and coordinated by the DCIC. The provided input was further curated and formalized by defining the controlled vocabularies, data formats, and by mapping to the external ontologies and repositories when applicable. As a result, the experimental metadata standards for the DDA, DIA, and targeted data were harmonized to further enable data integration and analysis of the LINCS-generated data. In addition, the LINCS proteomics assays have been described and maintained under the BioAssay Ontology (24).

The LINCS proteomics metadata standards describe different aspects of proteomics experiments such as (i) high-level metadata (e.g. instrument, software, software version, etc.), (ii) sample preparation, (iii) processing, (iv) fragmentation, (v) internal standards, and (vi) precursor information. The standards for sample preparation, processing, and fragmentation were completely aligned across all proteomics assays deployed by LINCS centers, while the experimental metadata standards related to the internal standards and precursor information were aligned for the DIA and targeted data when applicable. These extensive proteomics data descriptors may be very useful for other projects and research individuals to describe the proteomics data and we expect the adoption beyond the LINCS project. In fact, the LINCS experimental proteomics metadata standards were used as a starting point for the development of the metadata standards for proteomics data generated under the Illuminating Druggable Genome (IDG) project (25).

The annotated LINCS proteomics data is processed and released via the LINCS Data Portal (LDP) (https://lincsportal.ccs.miami.edu/dcic-portal) where the data and the metadata can be queried and retrieved directly on the website or via API. The data processing pipeline includes: (i) the data and metadata submission (in a programmatic manner from the repositories such as HMS DB or Panorama, or by direct data file transfer to the DCIC FTP site); (ii) data and metadata validation; (iii) standardization and aggregation; and (iv) public release via LDP (26).

The LINCS proteomics data related to this manuscript can be found under the following LDG-IDs: LDG-1444 (DToxS data), LDG-1285 (PCCSE P100 data), LDG-1284 (PCCSE GCP data), LDG-1456 (HMS LINCS data), and LDG-1298 (NeuroLINCS), and has also been registered under the ProteomeXchange (https://www.proteomexchange.org/) with the following IDs: PXD014791, PXD017458, PXD017459, PXD026581, PXD021497, respectively. The list of the datasets with the corresponding identifiers and links is shown in Table 1.

**Table 1:** LINCS proteomics datasets identifiers and links to the LINCS Data Portal and ProteomeXchange (PRIDE and Panorama repositories)

LINCS center	LINCS data portal	ProteomeXchange
DToxS	LDG-1444	PXD014791
PCCSE	LDG-1285	PXD017458
PCCSE	LDG-1284	PXD017459
HMS LINCS	LDG-1456	PXD026581
NeuroLINCS	LDG-1298	PXD021497

The LINCS proteomics data has been provided under the terms of the Creative Commons Attribution 4.0 International license (https://creativecommons.org/licenses/by/4.0/), which permits use, sharing, adaptation, distribution, and reproduction in any medium or format, as long as the appropriate credit is given to the original author(s) and the source, as defined in the LDP.

### Data Levels

The LINCS data levels were built upon the TCGA data level concepts and have been developed to conceptually harmonize data across all LINCS assays. All LINCS data types have a hierarchy defined from the raw data, to processed data, and to signatures. Although the number of data levels can differ for different assays and data types and the definitions can be different due to the processing pipelines, all LINCS proteomics data start with level 0 that corresponds to the raw data. The raw data represents the raw files as they are generated by the corresponding instruments. The raw data is further processed to the higher-level data (usually level 1 and level 2), normalized data (level 3), and signatures (level 4 and level 5 data). The definitions of the LINCS proteomics data levels have been described in detail in the corresponding publications by each of the LINCS proteomics centers (14, 17, 18, 19).

As the central focus of the LINCS program is to generate and collect high-level data and signatures, these data have been primarily released in the LDP. The raw and lower-level LINCS data has been deposited to external repositories (Panorama, Synapse, dbGaP, GEO, etc.). Links to the raw data can be found in the corresponding LDP dataset pages.

## Internet Resources for Accessing and Interacting with LINCS Proteomics Data and Metadata

Tools and resources generated by the LINCS proteomics group include data portals and computational tools to access, process and visualize the LINCS proteomics data. Being able to explore, interact with and analyze the diverse types of assays and data sets, generated by the LINCS proteomics group, requires a specialized set of tools, uniquely developed, or tailored for each assay type. Thus, the LINCS proteomics group has incorporated and created multiple methods and tools to address this diversity that together represent the integrated picture of the range of responses of human cells exposed to the perturbations. In the following subsections, the data gateways and tools are briefly described, and an example is provided to illustrate how the data can be explored and analyzed.

The LINCS Proteomics Landing Page (LPLP) (https://www.lincsproteomics.org/) is designed to be a starting point for the exploration of proteomic data and facilitates navigation through the LINCS proteomics data. This portal provides case-motivated search and query types, e.g. using chemical similarity to a query compound to find its LINCS analogs, or using the protein network neighborhood of a query gene/protein to identify potentially relevant genetic perturbations for which proteomics signatures are available. LPLP also provides several views of proteomics data that represent the relationship between measured protein entities as a snapshot of the biological state captured by a proteomics assay. In addition, LPLP provides links to other resources and tools developed as part of LINCS, as well as those developed by the proteomics community.

Computational methods and tools to identify and quantify peptides, proteins, and post-translational modifications (PTMs) that are captured in modern mass spectrometers evolve constantly to match technological and experimental advances. In the current context, studying multi-omics landscapes of cellular perturbations requires further improvement and integration of tools for downstream analysis, interpretation, and visualization of proteomics data sets, in particular those involving PTMs, to accelerate scientific discovery and maximize the impact of proteomics studies by connecting them better with biological knowledge across not only proteomics but also other Omics domains. Motivated by these challenges, including those stemming specifically from LINCS, the piNET server (1) has been developed as a versatile web platform to facilitate mapping, annotation, analysis, and visualization of peptide, PTM, and protein level quantitative data generated by either targeted, shotgun or other proteomics approaches. In particular, piNET provides a fast mapping from peptides (with PTMs) to proteins, integrates iPTMnet, PhosphoSitePlus, and Signore to provide a comprehensive mapping from PTM sites to modifying enzymes that target those sites, and finally from proteins (with PTMs) to pathways, as well as further mechanistic insights by connecting LINCS signatures of chemical and genetic perturbations via iLINCS (27) and LINCS Data Portal 2.0 (28), using both interactive and programmatic (API) access.

**Fig. 2:**
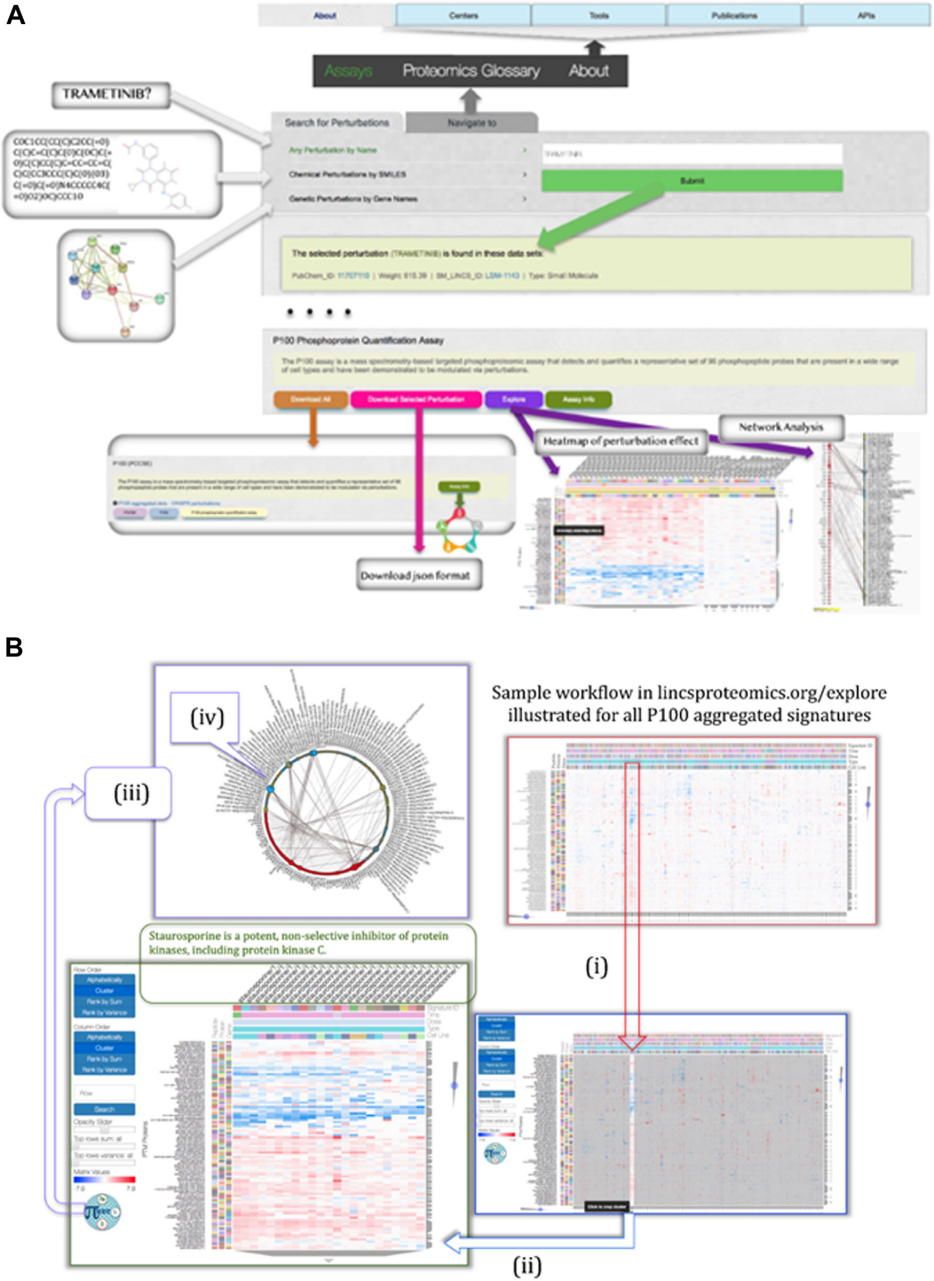
**Graphical illustration of exploring process in LINCS Proteomics Landing Page (LPLP)**. *A*, on the top, LPLP provides case motivated search and query types, e.g. using chemical similarity to a query compound in order to find its LINCS analogs, or using the protein network neighborhood of a query gene/protein in order to identify potentially relevant genetic perturbations for which proteomics signatures are available. On the *bottom*, the search query is directed to different sections and other tools and resources such as LINCS Data Portal. *B*, example showing the exploration of Staurosporine P100 signatures in LINCS Proteomics Landing Page. In this example, (i) P100 assay is shown as a cluster of samples where pronounced patterns in the peptide levels can be (ii) zoomed in, (iii) selected and enlarged, and the average signature of such selected profiles is then sent to piNET for further analysis. (iv) A circular phospho-network representation of the P100 assay is generated using known and predicted kinase-substrate relationships, illustrating a pattern of a lower abundance for multiple phosphopeptides targeted by kinases that are in turn inhibited by staurosporine, a non-specific kinase inhibitor.

To illustrate how LPLP can be used in conjunction with piNET to explore LINCS proteomics data we use here the example of a kinase inhibitor with a broad specificity profile, staurosporine. The effects of the perturbations’ concentrations and exposure times in multiple cell lines, in this case using the P100 assay to capture the underlying phosphostate of a cell, an interactive heatmap can be generated in LPLP to cluster proteomics signatures of interest and select specific clusters for further analysis. In this example, a cluster of samples with a pronounced pattern in the phosphopeptide levels is selected (step i in Fig. 2*B*), enlarged (step ii in Fig. 2*B*). Since piNET is integrated with LPLP, the average signature of such selected profiles can be then sent to piNET for further analysis by one simple click (step iii in Fig. 2*B*). A circular phospho-network representation of the P100 assay is generated using known and predicted kinase-substrate relationships (iv in the figure), illustrating a pattern of a lower abundance for multiple phosphopeptides targeted by kinases that are in turn inhibited by staurosporine, a non-specific kinase inhibitor.

## Integration of Multi-Modal LINCS Signatures

LPLP can be used as a starting point to explore small-molecule inhibitors and their targets by combining proteomics with transcriptomics and other modalities of data. In this example, we show how LINCS proteomics data can be used in conjunction with transcriptomics datasets in iLINCS and enrichment analysis in Enrichr (29, 30, 31) to explore LINCS data to identify putative kinase targets of small molecules.

To illustrate the approach, we started with all of the P100 signatures of chemical perturbations, clustered using Clustergrammer in the LPLP explore page (32). We agnostically explored if the phospho-proteomics signature of chemical perturbations can be used to identify the biological effects of the chemical perturbations. We first utilized cluster stability analysis for adequate cluster refinement. For the selection of cluster resolution, a subsampling was performed by randomly selecting 80% of the cells from the original dataset for 100 times. The Jaccard Index was computed as a measure of cluster stability by comparing clusters obtained from the original dataset with those obtained using 100 subsamples, similar to the previously published workflow (33).

**Fig. 3:**
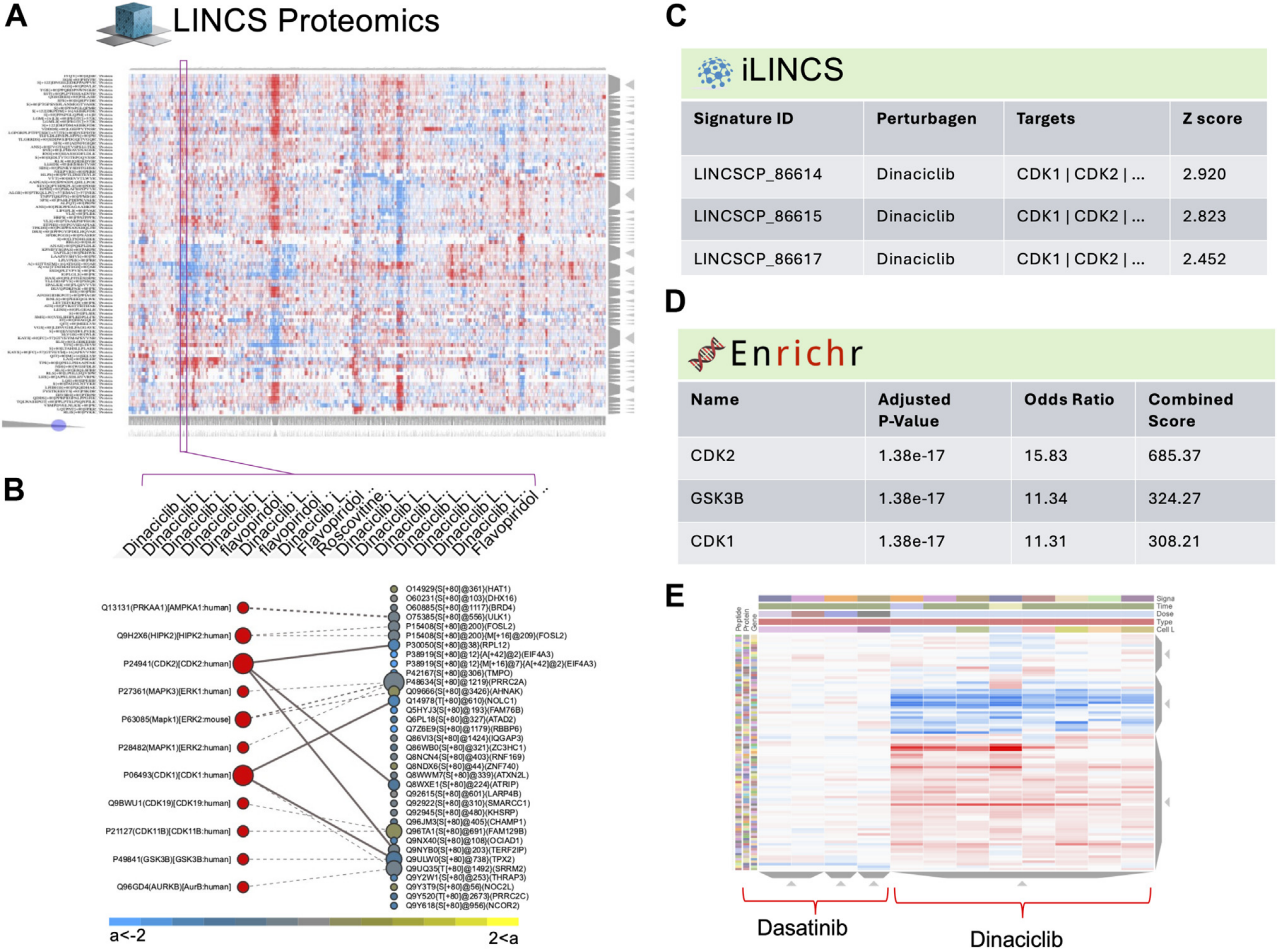
**Multimodal analysis of chemical perturbation signatures**. *A*, Selecting the most robust signature from LPLP P100 signatures, identifies two CDK inhibitors. *B*, Submission of the phospho-peptide signature of the selected cluster to piNET identifies CDK kinase as an active kinase. *C*, Dinaciclib was identified as a highly concordant signature to CDK gene knockdown signatures using iLINCS. *D*, Dinaciclib is suggested as an active Kinase using Enrichr enrichment analysis of CDK gene knockdown signatures. *E*, Dinaciclib shows a stronger phospho-peptide signal compared to Dasatinib (a non-specific kinase inhibitor).

After identifying the best cluster resolution, for the selection of specific clusters in the heatmap, we used a method that was previously described by Neftel *et al*. (34) We first standardized the peptide readouts, and the mean of the squares of these values were used to define a score for each signature. The cluster of interest is selected as the highest cluster score. The majority of perturbagens in such identified clusters comprise CDK inhibitors, including various doses of Dinaciclib and Flavopiridol, which are known CDK inhibitors (35) (Fig. 3*A*). The P100 signature of the cluster is analyzed using piNET to identify CDK1, CDK2, and other upstream kinases targeting differentially abundant phosphopeptides (Fig. 3*B*). To further investigate the connection between CDK1 and its inhibitor Dinaciclib, we explored the gene-knockdown transcriptional signatures of CDK1 in iLINCS. Here, Dinaciclib was identified as a highly concordant signature (Fig. 3*C*). This is further supported by Kinase Enrichment analysis of Dinaciclib signature using Enrichr (Fig. 3*D*). While proteomics analysis through piNET indicated downstream proteins affected by Dinaciclib, transcriptomics analysis through iLINCS and Enrichr presented a pathway-level view of the Dinaciclib signature. Finally, we compared Dinaciclib to another CDK inhibitor, Dasatinib. Dasatinib is less specific than Dinaciclib, targeting BCR-ABL, Src kinases, Btk tyrosine kinases, and other proteins in addition to CDKs. Consequently, Dinaciclib results in a stronger signal compared to Dasatinib (36, 37) when using the “Compare Perturbations” feature in LINCS proteomics (Fig. 3*E*).

## Summary

Unlike the other large-scale data generation efforts, the LINCS program employed a wide range of assay technologies cataloging diverse cellular responses. Here, we present an overview of the LINCS proteomics data and challenges to harmonize, annotate, and integrate the data. Although the four LINCS proteomics centers have produced unique and very diverse datasets and resources using state-of-the-art technologies, we have ensured that the data follows the FAIR principles. We overcame the challenges of harmonizing the various data and establishing formal specifications of the metadata to explicitly represent the biological and experimental aspects of the data. We have established the minimum metadata specifications and controlled vocabularies to sufficiently describe the data. The established metadata is critical to generating integrated and interpretable views of diverse LINCS proteomics results. To the best of our knowledge, this is a unique effort to harmonize and annotate such diverse proteomics data generated by different technology platforms and assays, and therefore the LINCS proteomics metadata standards specifications can be adopted beyond the LINCS data. The annotated LINCS proteomics data is organized, and publicly available to the community via the LINCS Data Portal, and all datasets can be freely downloaded and shared.

## Conflict of Interest

The authors declare the following financial interests/personal relationships which may be considered as potential competing interests.: R.L.G. is on the advisory board of ProtiFi, LLC, and receives no compensation of any kind.
