# Corrigendum to “Comprehensive Proteomics Metadata and Integrative Web Portals Facilitate Sharing and Integration of LINCS Multiomics Data”

**DOI:** 10.1016/j.mcpro.2025.100995

**Published:** 2025-08-07

**Authors:** Dušica Vidović, Behrouz Shamsaei, Stephan C. Schürer, Phillip Kogan, Szymon Chojnacki, Michal Kouril, Mario Medvedovic, Wen Niu, Evren U. Azeloglu, Marc R. Birtwistle, Yibang Chen, Tong Chen, Jens Hansen, Bin Hu, Ravi Iyengar, Gomathi Jayaraman, Hong Li, Tong Liu, Eric A. Sobie, Yuguang Xiong, Matthew J. Berberich, Gary Bradshaw, Mirra Chung, Robert A. Everley, Ben Gaudio, Marc Hafner, Marian Kalocsay, Caitlin E. Mills, Maulik K. Nariya, Peter K. Sorger, Kartik Subramanian, Chiara Victor, Maria Banuelos, Victoria Dardov, Ronald Holewinski, Danica-Mae Manalo, Berhan Mandefro, Andrea D. Matlock, Loren Ornelas, Dhruv Sareen, Clive N. Svendsen, Vineet Vaibhav, Jennifer E. Van Eyk, Vidya Venkatraman, Steve Finkbiener, Ernest Fraenkel, Jeffrey Rothstein, Leslie Thompson, Jacob Asiedu, Steven A. Carr, Karen E. Christianson, Desiree Davison, Deborah O. Dele-Oni, Katherine C. DeRuff, Shawn B. Egri, Alvaro Sebastian Vaca Jacome, Jacob D. Jaffe, Daniel Lam, Lev Litichevskiy, Xiaodong Lu, James Mullahoo, Adam Officer, Malvina Papanastasiou, Ryan Peckner, Caidin Toder, Joel Blanchard, Michael Bula, Tak Ko, Li-Huei Tsai, Jennie Z. Young, Vagisha Sharma, Jarek Meller, Michael J. MacCoss

MOLECULAR AND CELLULAR PROTEOMICS 24 (7) 2025 100947

The author list, author contributions, and the acknowledgments section for this paper have been corrected as follows:

Authors:

Dušica Vidović, Behrouz Shamsaei, Stephan C. Schürer, Phillip Kogan, Szymon Chojnacki, Michal Kouril, Mario Medvedovic, Wen Niu, Evren U. Azeloglu, Marc R. Birtwistle, Yibang Chen, Tong Chen, Jens Hansen, Bin Hu, Ravi Iyengar, Gomathi Jayaraman, Hong Li, Tong Liu, Eric A. Sobie, Yuguang Xiong, Matthew J. Berberich, Gary Bradshaw, Mirra Chung, Robert A. Everley, Ben Gaudio, Marc Hafner, Marian Kalocsay, Caitlin E. Mills, Maulik K. Nariya, Peter K. Sorger, Kartik Subramanian, Chiara Victor, Maria Banuelos, Victoria Dardov, Ronald Holewinski, Danica-Mae Manalo, Berhan Mandefro, Andrea D. Matlock, Loren Ornelas, Dhruv Sareen, Clive N. Svendsen, Vineet Vaibhav, Jennifer E. Van Eyk, Vidya Venkatraman, Steve Finkbiener, Ernest Fraenkel, Jeffrey Rothstein, Leslie Thompson, Jacob Asiedu, Steven A. Carr, Karen E. Christianson, Desiree Davison, Deborah O. Dele-Oni, Katherine C. DeRuff, Shawn B. Egri, Alvaro Sebastian Vaca Jacome, Jacob D. Jaffe, Daniel Lam, Lev Litichevskiy, Xiaodong Lu, James Mullahoo, Adam Officer, Malvina Papanastasiou, Ryan Peckner, Caidin Toder, Joel Blanchard, Michael Bula, Tak Ko, Li-Huei Tsai, Jennie Z. Young, Vagisha Sharma, Jarek Meller, Michael J. MacCoss

Author Contributions:

J. B., G. B., M. C., R. P., Y. X., C. T., M. J. B., M. H., R. A. E., B. G., D. L., L. L., J. D. J., A. O., T. L., M. P., E. A. S., X. L., J. M., H. L., M. B., V. D., M. B., K. S., C. V., J. Z. Y.; R. H., T. K., D. M., L. T., M. K. N., P. K. S., M. K., C. E. M., B. M., D. S., C. N. S., A. D. M., L. O., S. B. E., Y. C., T. C., A. S. V. J., D. O. D., E. U. A., K. C. D., M. R. B., R. I., G. J., J. H., B. H., J. R., L. T., K. E. C., D. D., S. F., V. V., J. E. V. E., E. F., J. A., S. A. C., and V. V. resources; M. J. M., J. M., P. K., S. C. S., and M. M. writing–review & editing; M. J. M., J. M., D. V., and B. S. writing–original draft; M. J. M., J. M., D. V., S. C. S., B. S., and M. M. conceptualization; P. K. formal analysis; V. S., S. C., B. S., M. K., M. M., S. C. S., B. S., M. M., and W. N. software; D. V., S. C. S., B. S., and M. M. methodology; D. V. data curation.

Acknowledgements:

This work was supported by the 10.13039/100000002National Institutes of Health (NIH) [U54HL127624: BD2K-LINCS DCIC; U54HG008098 and S10OD025047: DToxS; U54HL127365: HMS LINCS; U54NS091046: NeuroLINCS; U54HG008097: PCCSE]; 10.13039/100000092National Library of Medicine (NLM) [U01LM012630: BD2K, Enhancing the efficiency and effectiveness of digital curation for biomedical ‘big data’]. DV and SCS are also grateful for the National Center for Advancing Translational Sciences (NCATS) award [U24TR002278: Illuminating the Druggable Genome Resource Dissemination and Outreach Center, IDGRDOC] and National Human Genome Research Institute (NHGRI) award [U24HG012674: Molecular Phenotypes of Null Alleles in Cells (MorPhiC) Data Resource and Administrative Coordinating Center (DRACC)]. The authors sincerely thank Dr Ajay Pillai from the National Institutes of Health for his guidance and support for the conceptualization of this manuscript.

